# Incidence and prognostic impact of new-onset atrial fibrillation in patients with septic shock: a prospective observational study

**DOI:** 10.1186/cc9057

**Published:** 2010-06-10

**Authors:** Rainer Meierhenrich, Elisa Steinhilber, Christian Eggermann, Manfred Weiss, Sami Voglic, Daniela Bögelein, Albrecht Gauss, Michael Georgieff, Wolfgang Stahl

**Affiliations:** 1 Department of Anesthesiology, University of Ulm, Prittwitzstr. 43, 89075 Ulm, Germany

## Abstract

**Introduction:**

Since data regarding new-onset atrial fibrillation (AF) in septic shock patients are scarce, the purpose of the present study was to evaluate the incidence and prognostic impact of new-onset AF in this patient group.

**Methods:**

We prospectively studied all patients with new-onset AF and all patients suffering from septic shock in a non-cardiac surgical intensive care unit (ICU) during a 13 month period.

**Results:**

During the study period, 687 patients were admitted to the ICU, of which 58 patients were excluded from further analysis due to pre-existing chronic or intermittent AF. In 49 out of the remaining 629 patients (7.8%) new-onset AF occurred and 50 out of the 629 patients suffered from septic shock. 23 out of the 50 patients with septic shock (46%) developed new-onset AF. There was a steady, significant increase in C-reactive protein (CRP) levels before onset of AF in septic shock patients. ICU mortality in septic shock patients with new-onset AF was 10/23 (44%) compared with 6/27 (22%) in septic shock patients with maintained sinus rhythm (SR) (*P *= 0.14). During a 2-year follow-up there was a trend towards an increased mortality in septic shock patients with new-onset AF, but the difference did not reach statistical significance (*P *= 0.075). The median length of ICU stay among surviving patients was longer in patients with new-onset AF compared to those with maintained SR (30 versus 17 days, *P *= 0.017). The success rate to restore SR was 86%. Failure to restore SR was associated with increased ICU mortality (71.4% versus 21.4%, *P *= 0.015).

**Conclusions:**

AF is a common complication in septic shock patients and is associated with an increased length of ICU stay among surviving patients. The increase in CRP levels before onset of AF may support the hypothesis that systemic inflammation is an important trigger for AF.

## Introduction

Cardiac arrhythmias are well-known complications in postoperative and critically ill patients. In the past, the main concern has been focused on arrhythmias after cardiac and noncardiac thoracic surgery. Following coronary artery bypass grafting, the reported incidence of atrial arrhythmias range from 10 to 65% [1,2]. Following noncardiac thoracic surgery, the incidence of atrial arrhythmias range from 9 to 29% and was associated with a higher ICU admission rate, longer hospital stay and greater 30-day mortality [3].

In recent years increasing attention has been devoted to atrial arrhythmias after noncardiac, nonthoracic surgery [4-6]. Brathwaite and colleagues pointed out a high incidence (10%) of new-onset atrial arrhythmias in patients undergoing major non-cardiothoracic surgery [6]. Seguin and colleagues focused on new-onset atrial fibrillation and observed an incidence of 5% on a noncardiac surgical ICU [5]. Both working groups demonstrated that new-onset atrial arrhythmias in this patient group are associated with increased morbidity and mortality [5,6]. In agreement with former studies, Seguin and colleagues identified sepsis and septic shock as a risk factor for the development of new-onset atrial fibrillation (AF) [5,7].

Interestingly, apart from the results presented by Seguin and colleagues [5], who included a subgroup of 23 patients with septic shock, no further prospectively acquired data about the incidence and prognostic impact of new-onset AF in patients with septic shock are available.

Therefore, the main purpose of the present study was to assess the incidence of new-onset AF in patients with septic shock, admitted on a noncardiac surgical ICU, and to evaluate its prognostic impact with respect to mortality and length of ICU stay.

Further, there is increasing suspicion that inflammation *per se *is a main trigger for the development and maintenance of AF. Therefore, we analyzed inflammation parameters before and after occurrence of new-onset AF.

Finally, no data regarding the treatment of new-onset AF in critically ill patients are available to date. Thus, we describe the success rate to restore sinus rhythm (SR) using antiarrhythmic drugs and electrical cardioversion in this patient group.

## Materials and methods

The study was performed on a general surgical 14-bed ICU, including thoracic but not cardiac surgery, over a 13 month-period (March 2006 to March 2007). This ICU admits trauma patients and all types of postoperative surgical patients, including those with neurologic, lung and vascular surgery, except cardiac surgery, who require mechanical ventilation, renal replacement therapy, hemodynamic support, or special observation. The study was approved by the ethics committee of the University of Ulm (Approval No 23/06) and informed consent was obtained from all patients who were conscious during inclusion as well as those patients who regained consciousness during the follow-up.

### Study design

The study was designed as a prospective single-center observational study. During the study period, all patients who developed new AF on the ICU and all patients fulfilling the criteria of septic shock were included in this study. Patients with known intermittent AF or episodes of AF in their history and patients with chronic AF were registered but not included in the study. The majority of patients were examined preoperatively by an anesthesiologist from our clinic. In case of clinical signs for coronary artery disease (e.g. angina pectoris) or heart failure, patients were routinely examined by a cardiologist and in the first step an exercise electrocardiogram and transthoracic echocardiogram were performed. The following variables were recorded for all included patients: sex, age, premorbidity including cardiovascular diseases (hypertension, coronary artery disease, heart failure, cardiomyopathy, valvular disease, previous arrhythmias) and chronic obstructive pulmonary disease. Previous regular medication was also documented including ß-blockers, digitalis glycosides, calcium channel inhibitors and angiotensin-converting enzyme inhibitors.

When AF occurred, current clinical variables including mechanical ventilation, use and dosage of catecholamines, serum electrolytes (Na^+^, K^+^, Ca^2+^), and renal replacement therapy were registered. Furthermore, in all patients with new-onset AF, the number of leucocytes, C-reactive protein (CRP) and maximum daily temperature were recorded - retrospectively if possible during the three days before onset of AF and prospectively for the following five days after onset of AF. The Simplified Acute Physiologic Score II (SAPS II) [8] on admission as well as the daily calculated Sequential Organ Failure Assessment (SOFA) score [9] were determined in all patients. Moreover, length of stay in the ICU and ICU-mortality were documented. All patients were followed-up for two years after admission to the ICU.

#### Diagnosis of new-onset atrial fibrillation

In all patients admitted to the ICU, a continuous three-lead electrocardiogram was registered. In case of sudden increase in heart rate (> 110 beats/min) or loss of interval between one R wave and the next R wave (RR-interval) regularity, a 12-lead electrocardiogram was derived. The diagnosis of AF was then made if irregular ventricular activity and chaotic atrial activity with no apparent P waves were present [10].

#### Treatment of new-onset atrial fibrillation

All patients with new-onset AF received treatment to re-establish SR consisting of either electrical cardioversion or medical therapy (amiodarone, ß-blockers, digitalis glycosides), or a combination of these approaches. Treatment of new-onset AF was not performed according to a fixed protocol, but according to the decision of the responsible intensivist. Type of AF therapy and success of the therapy with respect to restoration of SR were recorded in all patients.

#### Diagnosis of septic shock

The diagnosis of septic shock was based on the definitions of the American College of Chest Physicians/Society of Critical Care Medicine Consensus Conference [11]. The presence of the following criteria were required for the diagnosis of septic shock: (i) systemic inflammatory response syndrome; (ii) evidence of infection; (iii) organ dysfunction; (iiii) circulatory failure requiring vasopressor therapy with norepinephrine for (> 0.1 μg/kg/min) more than five hours to maintain mean arterial blood pressure above 65 mmHg despite adequate volume substitution.

### Statistical analysis

For continuous variables, the median and range are reported, whereas for categorical variables, the number of patients in each category and the corresponding percentage are given. The characteristics of different groups were compared using the exact Mann-Whitney U-test for continuous variables and Fisher's-exact test for categorical variables. Changes of CRP plasma levels, number of leucocytes and maximum daily temperature over time were analyzed by one-way analysis of variance, and, if significant, Dunnett's method was used to compare the variables with the baseline value (value observed three days before onset of AF).

The Kaplan-Meier method was used to create the survival curves for septic shock patients with new-onset AF and for septic shock patients with maintained SR. The survival curves were compared using the log-rank test.

For all analyses, a *P*-value of less than 0.05 was considered to be significant.

## Results

### Overall occurrence of new-onset AF

A total of 687 patients were admitted to the ICU during the study period. Of these 687 patients, 58 revealed pre-existing chronic or intermittent atrial AF. Forty-nine (7.8%) of the remaining 629 patients developed new-onset AF during their stay on the ICU. The incidence of new-onset AF was 9.2% (38/413) in men and 5.1% (11/216) in women; the difference was statistically not significant (*P *= 0.10). In 67% of patients, new-onset AF occurred within the first three days of ICU stay.

### Occurrence of septic shock and incidence of AF in septic shock

Sixty-four of all admitted patients (9.1%) suffered from septic shock. Fourteen of the 64 patients with septic shock had pre-existing chronic AF. Remarkably, of the remaining 50 patients with septic shock, 23 (46%) developed new-onset AF.

On the other hand, in only 26 of 579 (4.5%) patients without septic shock did new-onset AF occur. Thus, new-onset AF was much more frequent in patients with septic shock than in those without septic shock (46% versus 4.5%; *P *< 0.001).

A comparison of septic shock patients with maintained SR versus those with new-onset AF is given in Tables 1 and 2 (*P*_2_-value). Septic shock patients with new-onset AF were older (*P *< 0.01) and more frequently suffered from arterial hypertension (*P *= 0.02).

**Table 1:** Patient characteristics

	New-onset AF, no septic shock (n = 26)	New-onset AF and septic shock (n = 23)	Maintained SR and septic shock (n = 27)	*P*_1_-value	*P*_2_-value
Sex (f/m)	6/20	5/18	12/15	1.00	0.14
Age (years)	67 (46-84)	66 (41-85)	56 (18-80)	0.59	< 0.01
History of hypertension	16	17	11	0.38	0.02
Coronary artery disease	3	5	2	0.45	0.23
Heart failure	0	1	0	0.47	0.46
Valvular disease	0	1	1	0.47	1.00
COLD	4	4	1	1.00	0.17
**Premedication**					
β-blocker	8	8	6	1.0	0.36
digitalis	0	1	0	0.47	0.46
Calcium antagonist	5	7	2	0.51	0.06
ACE inhibitor	10	2	7	0.02	0.15
**Type of surgery**					
Lung surgery	4	4	2	-	-
Abdominal surgery	11	12	19	-	-
Neurosurgery	4	1	1	-	-
Traumatologic surgery	1	0	2	-	-
Vascular surgery	1	0	2	-	-
Aortic surgery	5	4	1	-	-
Others	0	2	0	-	-

Data are given as median (range in parenthesis) or as number. *P*_1_-value, patients with new-onset atrial fibrillation (AF) without septic shock vs. septic shock patients with new-onset AF; *P*_2_-value, septic shock patients with new-onset AF vs. septic shock patients with maintained sinus rhythm.

ACE, angiotensin-converting enzyme; COLD, chronic obstructive lung disease; f, female; m, male; SR, sinus rhythm.

**Table 2:** Severity of illness scores, laboratory tests and use of catecholamines during ICU stay

	New-onset AF, no septic shock (n = 26)	New-onset AF and septic shock (n = 23)	Maintained SR and septic shock (n = 27)	*P*_1_-value	*P*_2_-value
**Severity of illness scores**					
SOFA max	8.5 (4-14)	12 (7-17)	9 (5-18)	< 0.01	0.01
SAPS II	34 (7-60)	31 (15-63)	30 (12-65)	0.87	0.12
**Max. CRP level (mg/dl)**	249 (14-339)	288 (72-483)	273 (23-412)	0.04	0.28
**Use of catecholamines**					
Noradrenaline max. (μg/kg/min)	0.18 (0.00-1.00)	0.50 (0.15-2.00)	0.30 (0.15-1.40	< 0.01	0.13
Noradrenaline at AF (μg/kg/min)	0.05 (0.00-0.40)	0.40 (0.03-1.10)	-	< 0.01	
Dobutamine (number of patients)	2	10	6	< 0.01	0.14
**Serum electrolytes at AF**					
K^+ ^(mval/l)	4.4 (3.9-5.0)	4.4 (3.8-5.6)	-	0.63	
Na^+ ^(mval/l)	137 (130-163)	140 (132-161)	-	0.42	
Ca^++ ^(mval/l)	1.2 (1.1-1.6)	1.1 (0.7-1.3)	-	0.10	

Data are given as median (range in parenthesis) or number. *P*_1_-value, patients with new-onset atrial fibrillation (AF) without septic shock vs. septic shock patients with new-onset AF; *P*_2_-value, septic shock patients with new-onset AF vs. septic shock patients with maintained sinus rhythm (SR).

SOFA max, maximum of the daily calculated sequential organ failure assessment score; SAPS II, simplified acute physiologic score II on admission; Max. CRP level, maximal C-reactive protein level during ICU stay; noradrenaline max, maximal noradrenaline dose during ICU stay; noradrenaline at AF, noradrenaline dose when AF occurred; serum electrolytes at AF, serum electrolyte concentrations when AF occurred.

Septic shock patients with new-onset AF demonstrated a significantly higher maximal SOFA score during the ICU stay compared with septic shock patients with maintained SR (*P *= 0.01), although the SAPS II score at ICU admission was not significantly different (Table 2). Doses of noradrenaline and frequencies of dobutamine use did not significantly differ between septic shock patients with new-onset AF versus those with maintained SR (Table 2). Serum electrolyte levels did not reveal apparent disturbances when new-onset AF occurred (Table 2).

### Inflammation parameters before and after onset of AF

**Figure 1:**
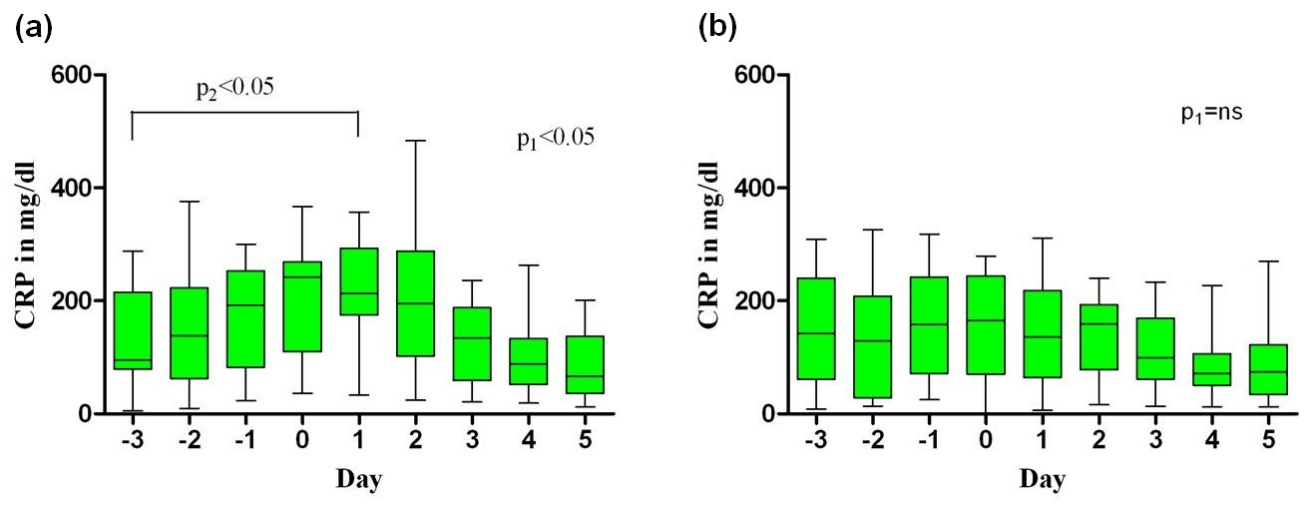
**Time course of CRP plasma concentrations before, during and after onset of new AF**. **(a) **Patients with new-onset atrial fibrillation (AF) and septic shock. **(b) **Patients with new-onset AF without septic shock. The median, interquartile range (box), minimum and maximum are shown. Day 0, day of occurrence of AF; Day -3, three days before new-onset of AF; Day 5, five days after occurrence; *P*_1_-value, analysis of variance (ANOVA) over time; *P*_2_-value, comparison of C-reactive protein (CRP) levels Day 1 versus CRP levels Day -3 (Dunnett's method). (b) Note: *P*_2_-value was not calculated for patients with new-onset AF without septic shock as ANOVA did not demonstrate significant change over time.

CRP plasma levels over time are shown for AF patients with septic shock and AF patients without septic shock in Figures 1a and 1b. Both groups demonstrated high median CRP plasma levels when new-onset AF occurred (242 versus 165 mg/dl). AF patients with septic shock revealed a continuous increase in CRP plasma levels before occurrence of AF (Figure 1a). Maximal CRP plasma levels observed during ICU stay did not differ between septic shock patients with new-onset AF and those who maintained SR (Table 2).

Also, the maximum daily temperature revealed a slight increase up to the first day after new-onset AF, whereas the number of leucocytes demonstrated a slight decrease, but these changes were statistically not significant (data not shown).

### Outcome

**Figure 2:**
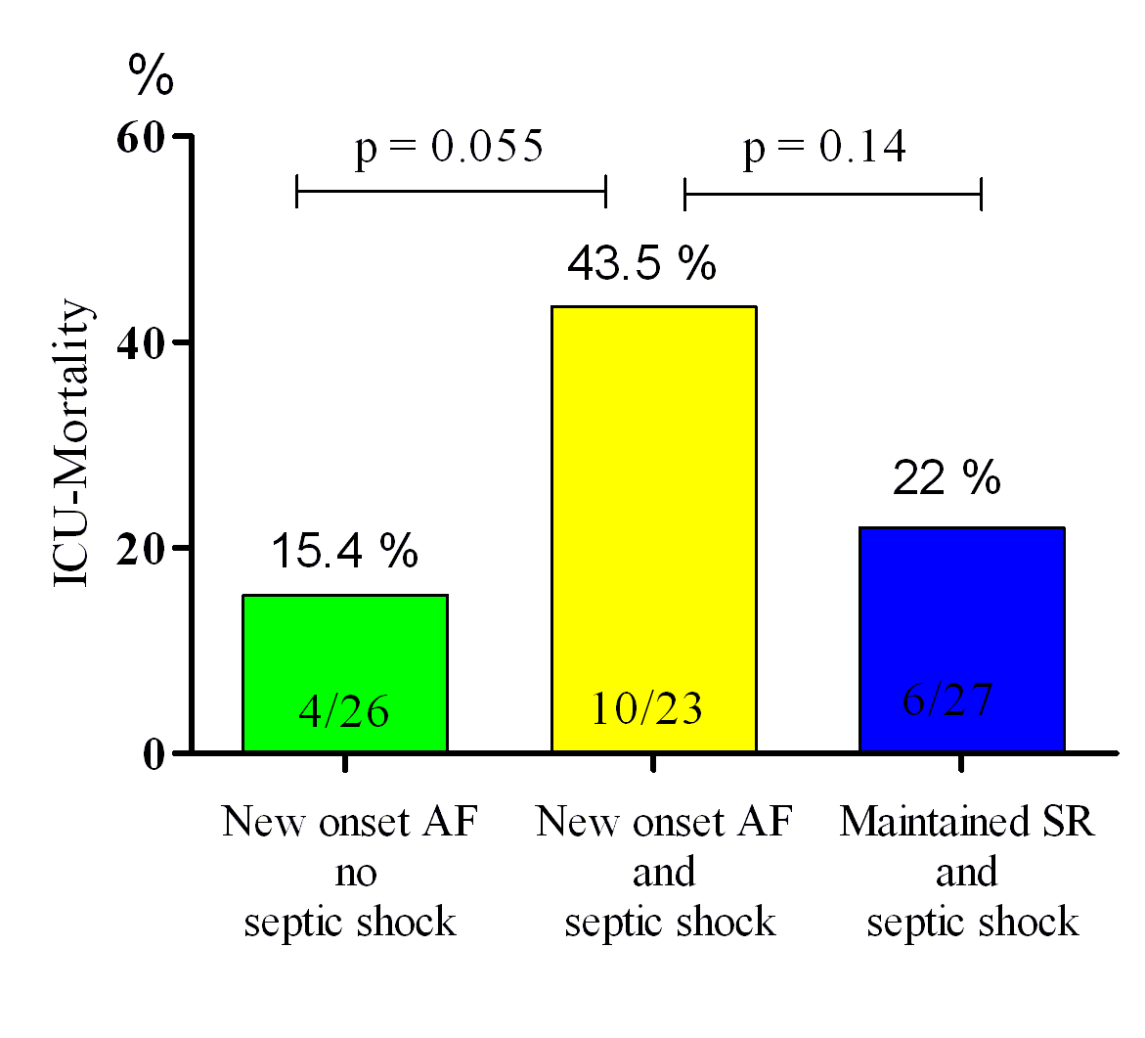
**ICU mortality**. AF, atrial fibrillation; SR, sinus rhythm.

ICU mortality rate in septic shock patients with new-onset AF was 10 out of 23, compared with 6 out of 27 in septic shock patients who maintained SR. This difference did not reach statistical significance (*P *= 0.14). Mortality rate in AF patients without septic shock was 4 out of 26 (Table 3 and Figure 2).

**Table 3:** Patients outcome

	New-onset AF, no septic shock (n = 26)	New-onset AF and septic shock (n = 23)	Maintained SR and septic shock (n = 27)	*P*_1_-value	*P*_2_-value
ICU-mortality	4 (15%)	10 (44%)	6 (22%)	0.06	0.14
28-day mortality	4 (15%)	9 (39%)	6 (22%)	0.10	0.22
60-day mortality	6 (23%)	11 (48%)	7 (26%)	0.08	0.14
ICU length of stay, days (surviving patients)	10.5 (2-45) (n = 22)	30 (9-123) (n = 13)	17 (4-48) (n = 21)	< 0.001	0.017

Data are given as number (percentage) or median (range in parenthesis). *P*_1_-value, patients with new-onset AF without septic shock versus septic shock patients with new-onset atrial fibrillation (AF); *P*_2_-value, septic shock patients with new-onset AF versus septic shock patients with maintained sinus rhythm (SR).

**Figure 3:**
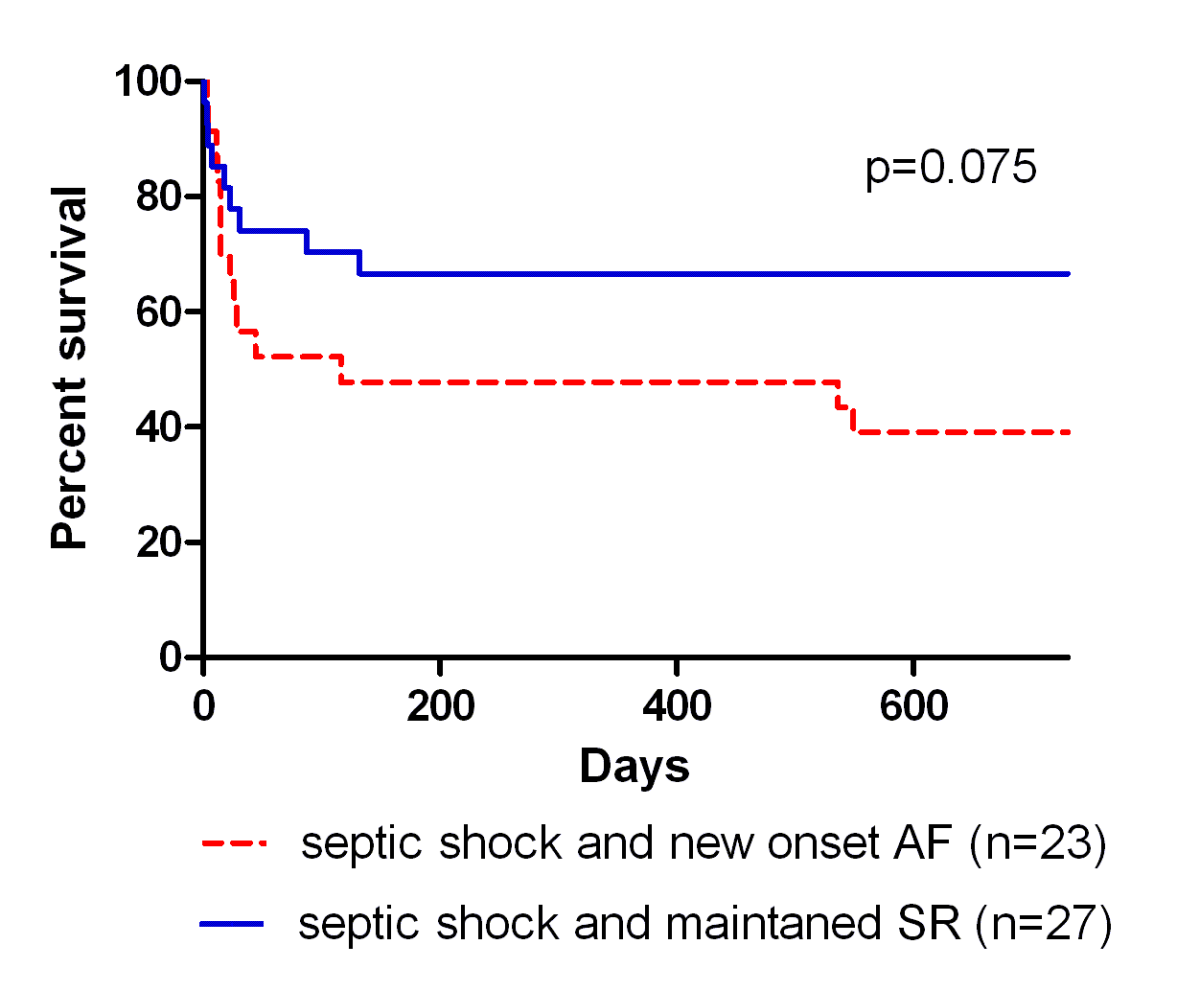
**Kaplan-Meier survival curves for septic shock patients with new-onset atrial fibrillation and septic shock patients with maintained sinus rhythm**. AF, atrial fibrillation; SR, sinus rhythm.

Mortality rates at 28 and 60 days after ICU admission are given in Table 3. The Kaplan-Meier curves, calculated on the basis of a two-year follow-up, are shown in Figure 3. There was a trend towards an increased mortality in septic shock patients with new-onset AF compared with septic shock patients with maintained SR, but the difference was statistically not significant (*P *= 0.075).

**Figure 4:**
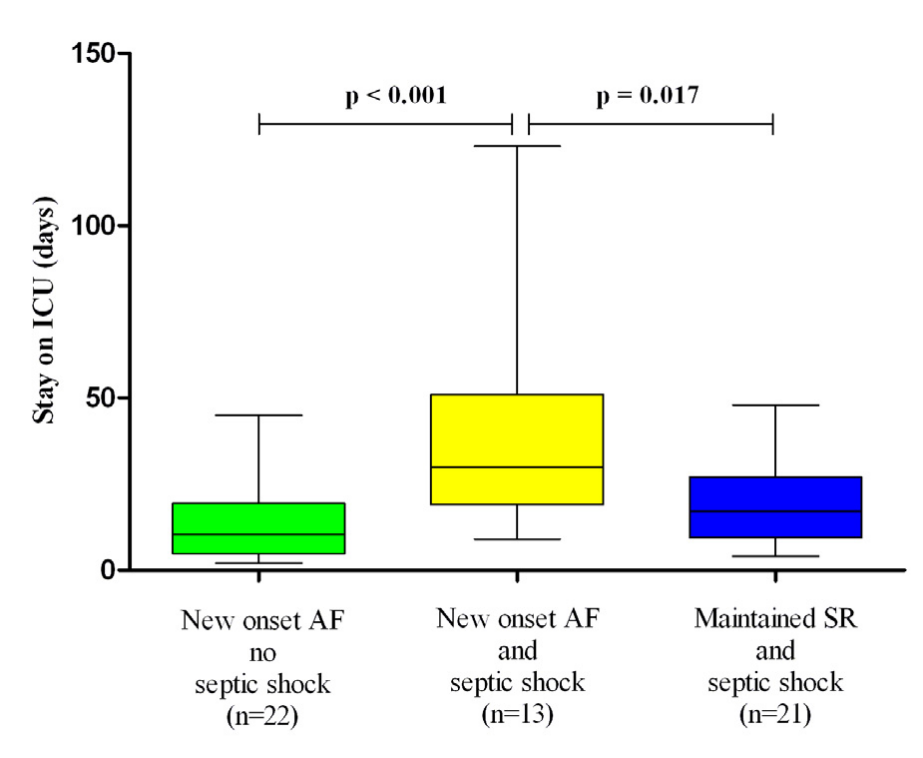
**ICU length of stay of surviving patients**. The median, minimum, maximum and interquartile range (box) are shown. AF, atrial fibrillation; SR, sinus rhythm.

Among surviving patients, those with septic shock and new-onset AF had a longer stay on the ICU (median stay 30 days) than those with septic shock and maintained SR (median stay 17 days, *P *= 0.017) and those with new-onset AF without septic shock (median stay 11 days, *P *< 0.001; Figure 4).

### Success rate to restore SR and recurrence of AF

Electrical cardioversion was performed in 17 of 49 patients with AF, but was combined in all cases with additional drug therapy. Amiodarone was the drug used most frequently (36/49 patients), followed by digitalis glycosides (31/49) and ß-blockers (25/49), indicating that the majority of patients received a combination of antiarrhythmic drugs.

In 42 out of the 49 patients with new-onset AF, SR was successfully reconstituted, including 23 out of 26 patients without septic shock and 19 out of 23 patients with septic shock. Only one of the seven patients, who could not be converted to SR, did not receive amiodarone.

Failure to restore SR was associated with an increased ICU mortality. ICU mortality was 5 out of 7 patients who could not be cardioverted to SR in contrast to 9 out of 42 successfully cardioverted patients (*P *= 0.015).

Recurrence rate of AF was high (42.9%), with no significant difference between AF in patients with septic shock and AF patients without septic shock (48% versus 38%, *P *= 0.57).

## Discussion

In this prospective observational study, we demonstrate a high incidence of new-onset AF in septic shock patients. Remarkably, 46% of all patients with septic shock developed new-onset AF. Among surviving septic shock patients, those who developed new-onset AF had a prolonged ICU stay in comparison to septic shock patients with maintained SR. Further, septic shock patients with new-onset AF may have a poorer prognosis. In the present study, we found a trend towards an increased mortality during a two-year follow-up, but the difference was not statistically significant.

Overall, incidence of new-onset AF in our study was 7.8% (49/629), which is in the range of previous studies (1.8 to 10%) performed in noncardiac ICUs [5-7,12-14]. However, many of these studies did not clearly focus on AF but rather on a broad variety of atrial arrhythmias. Moreover, in older studies, the patients were not continuously monitored [12,13]. Seguin and colleagues exclusively looked at AF on a surgical ICU and found an incidence of new-onset AF of 5.3% [5]. Thus, our study confirms that new-onset AF is a common complication in critically ill patients. In agreement with previous studies, we found that in two-thirds of the patients, new-onset AF occurred within the first three days on the ICU [5,6].

Salman and colleagues retrospectively analysed patients with sepsis and reported an incidence of AF of 31% [15]. With respect to the incidence of new-onset AF in septic shock, Seguin and colleagues included a subgroup of 23 patients and observed new-onset AF in 30%, which is slightly lower when compared with our finding of 46% [5]. One reason for this difference might be our restrictive definition of septic shock, in particular the requirement of vasopressor therapy with norepinephrine for more than five hours with a dosage more than 0.1 μg/kg/min.

In the present study, septic shock patients with new-onset AF were older, more frequently revealed a history of hypertension and developed a higher maximal SOFA score during ICU stay in comparison to septic shock patients with maintained SR. Age and a history of hypertension have been identified in previous studies as risk factors for the development of AF in non-selected ICU patients [5,7]. The higher SOFA score in septic shock patients with new-onset AF indicates that presumably there is an association between severity of illness and the development of AF.

A variety of further factors including pre-existing heart failure, ischemic heart disease, valvular disease, electrolyte disturbances and use of catecholamines have been addressed as potential co-factors or causes for the development of new-onset AF in critically ill patients [5-7]. In the current study, only a small number of patients developing new-onset AF revealed pre-existing heart failure, ischemic heart disease or valvular disease. Furthermore, we did not find apparent electrolyte disturbances when new-onset AF occurred. Also, regarding the treatment with catecholamines there was no significant difference between septic shock patients with new-onset AF in comparison to those with maintained SR. The present data do not support the hypothesis that one of these factors plays a mayor role in the development of AF in critically ill patients.

The pathophysiological mechanism underlying the development of AF in critically ill patients and in particular in septic shock is not known. However, there is increasing evidence that the systemic inflammatory response *per se *is a predominant trigger of AF in critically ill patients. The occurrence of AF after cardiac surgery has been shown to be closely related to the complement CRP activation on the postoperative day two or three [16]. Also, in the non-operative setting, a series of studies has now demonstrated an association of elevated CRP levels with the development and maintenance of AF [17-19]. Chung and colleagues found two-fold higher CRP levels in patients with AF than in control subjects. Furthermore, patients with persistent AF had higher CRP levels than those with paroxysmal AF, suggesting that inflammation plays an important role in the maintenance of AF [17]. In addition, elevated CRP levels have been correlated to a decreased success rate of electrical cardioversion and subsequent maintenance of SR [20-22]. The hypothesis, that inflammation may trigger the development of AF in critically ill patients, is supported by our observation of increasing CRP plasma concentrations before the onset of AF in septic shock patients. Also, in AF patients without septic shock, CRP levels were very high when AF occurred. However, maximal CRP levels occurring during ICU stay did not differ between septic shock patients with new-onset AF and septic shock patients who maintained SR, indicating that other factors may contribute to the development of AF in critically ill patients.

Although new-onset of AF, as reemphasized by the present data, is a frequent and major problem in ICU patients, no evidence-based data regarding the treatment of AF for this patient group are available. In the current study, restoration of SR was possible in 85% of the patients. In the majority of patients, amiodarone was used, but was frequently combined with electrical cardioversion or other drugs. On the other hand, in 12 patients, restoration of SR was possible without the use of amiodarone. Although amiodarone seems to be an effective drug for restoration of SR, we do not know whether the outcome is positively affected by this measure. Previous studies on AF in non-critically ill patients have impressively demonstrated that restoration of SR patients does not automatically imply an improvement in clinical outcome [23,24]. Furthermore, prophylactic intravenous administration of amiodarone for supraventricular tachyarrhythmias after pulmonary surgery has been associated with an increased risk for the development of acute respiratory distress syndrome [25]. Therefore, prospective randomized controlled studies are necessary to evaluate the use of amiodarone in critically ill patients.

The present study contains several limitations. Presumably, the number of patients was too low to demonstrate a significant association between new-onset AF and mortality rate in septic shock patients. Further, due to the limited number of patients, it was not possible to perform a multivariate analysis to identify independent risk factors for the development of AF in septic shock patients. Moreover, therapy of AF was not performed according to a fixed protocol. Therefore, the failure rate to restore SR has to be appraised with caution.

## Conclusions

We have found that new-onset AF is a very common complication in septic shock patients that is associated with an increased ICU length of stay among surviving patients. Higher SOFA scores observed in septic shock patients with new-onset AF may indicate an association between severity of illness and the occurrence of AF. The observation of increasing CRP levels before onset of AF may support the hypothesis that systemic inflammation is an important trigger for the development of AF in critically ill patients. Success rate to restore SR by antiarrhythmic drugs and electrical cardioversion was high, and failure to restore SR was associated with increased mortality.

## Key messages

• Almost half of all patients with septic shock develop new-onset AF.

• New-onset AF in septic shock patients is associated with increased ICU length of stay among surviving patients.

• Septic shock patients with new-onset AF demonstrate a higher maximum SOFA score during ICU stay compared with those with maintained SR.

• Increasing CRP levels before onset of AF support the hypothesis that inflammation is an important trigger for the development of AF.

• Failure to restore SR in critically ill patients is associated with an increased mortality.

## Abbreviations

AF: atrial fibrillation; CRP: C-reactive protein; SAPS: Simplified Acute Physiologic Score; SOFA: Sequential Organ Failure Assessment; SR: sinus rhythm.

## Competing interests

The authors declare that they have no competing interests.

## Authors' contributions

RM contributed to design, data acquisition, statistical analysis and drafted the manuscript. CE contributed to data acquisition and drafted the manuscript. ES contributed to data acquisition, data analysis and presentation. MW contributed to data analysis and manuscript drafting. SV participated in data acquisition and statistical analysis. DB performed the long-term follow-up. AG contributed to data analysis and manuscript drafting. MG contributed to study design and manuscript drafting. WS contributed to data acquisition; statistical analysis and manuscript drafting. All authors read and approved the final manuscript.
